# Multimodal cortical neuronal cell type classification

**DOI:** 10.1007/s00424-024-02923-2

**Published:** 2024-02-20

**Authors:** Xiaoyi Mao, Jochen F. Staiger

**Affiliations:** Institute for Neuroanatomy, University Medical Center Göttingen, Georg-August-University, Kreuzbergring 36, 37075 Göttingen, Germany

**Keywords:** Cerebral cortex, Neuronal cell types, Excitatory neurons, Inhibitory neurons, Transcriptomics, Multimodal classification

## Abstract

Since more than a century, neuroscientists have distinguished excitatory (glutamatergic) neurons with long-distance projections from inhibitory (GABAergic) neurons with local projections and established layer-dependent schemes for the ~ 80% excitatory (principal) cells as well as the ~ 20% inhibitory neurons. Whereas, in the early days, mainly morphological criteria were used to define cell types, later supplemented by electrophysiological and neurochemical properties, nowadays. single-cell transcriptomics is the method of choice for cell type classification. Bringing recent insight together, we conclude that despite all established layer- and area-dependent differences, there is a set of reliably identifiable cortical cell types that were named (among others) intratelencephalic (IT), extratelencephalic (ET), and corticothalamic (CT) for the excitatory cells, which altogether comprise ~ 56 transcriptomic cell types (t-types). By the same means, inhibitory neurons were subdivided into parvalbumin (PV), somatostatin (SST), vasoactive intestinal polypeptide (VIP), and “other (i.e. Lamp5/Sncg)” subpopulations, which altogether comprise ~ 60 t-types. The coming years will show which t-types actually translate into “real” cell types that show a common set of multimodal features, including not only transcriptome but also physiology and morphology as well as connectivity and ultimately function. Only with the better knowledge of clear-cut cell types and experimental access to them, we will be able to reveal their specific functions, a task which turned out to be difficult in a part of the brain being so much specialized for cognition as the cerebral cortex.

## General introduction

The mammalian brain is a complex organ which comprises billions of neurons that altogether were recently suggested to consist of several thousand types of neurons [28]. The neurons of the cerebral cortex collectively shape the computations within networks that represent mental activities and govern behavior. The most practical means of navigating this complexity and thus unraveling the mysteries of neuronal diversity that contribute to cortical circuit structure and function is by classifying neurons into distinct types [39–41]. Therefore, the classification of neuronal cell types has been recognized as the pivotal pursuit within the realm of neuroscience research.

Historically, by means of histological stains, pioneers like Ramon y Cajal and Lorente de Nó relied on morphological criteria to identify cortical cell types [39]. In recent decades, the field has witnessed a transformative shift toward more sophisticated methods. Electrophysiological techniques brought functional insights into the classification, while neurochemical markers provided additional layers of characterization. However, it was the groundbreaking development of single-cell transcriptomics that overhauled laborious and low-throughput conventional approaches and revolutionized our ability to profile individual neurons at the most basic genetic level. This approach enables the identification of previously unknown cell types, revealing their unique genetic signatures and paving the way for a more comprehensive and nuanced classification of cortical neuronal cell types [40, 41].

Although strong consistency is observed among categories established through morphological, molecular, and physiological criteria [9, 26, 36], no single approach exists to fully encompass the inherently multimodal attributes of cell phenotypes and establish a universal standard for classification. In this review, we will mainly use the mouse brain as an example to shed light on the current status of cortical cell type classification.

## Current status of excitatory cell type classification

**Fig. 1:**
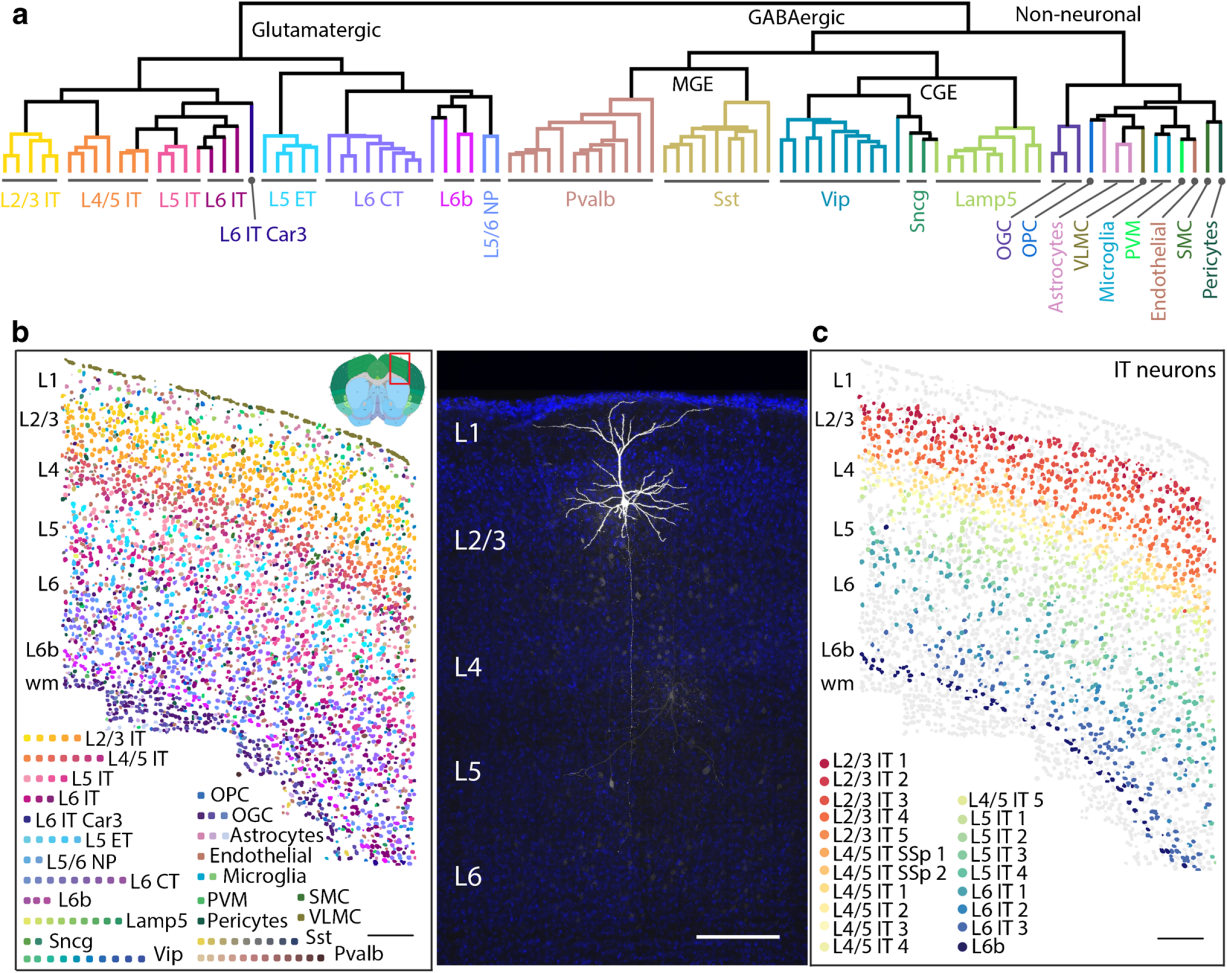
Comprehensive account of cortical cell types exemplified in the mouse primary motor cortex (MOp) by cluster analysis and spatial transcriptomics (MERFISH). **a** Glutamatergic, GABAergic neuronal and non-neuronal cells cluster dendrogram giving an overview of major groups and cell types, which are colored for their identity. Please note that GABAergic neurons not only cluster by major molecular markers but also by developmental origin (MGE: medial ganglionic eminence; CGE: caudal ganglionic eminence). **b** Spatial distribution of cell types identified by clustering (in **a**) shown with the same color code by spatial transcriptomics. Please note that due to layer identity being a major factor for the majority cell type of pyramidal neurons, a laminar pattern becomes apparent. Here, a section through MOp is displayed whose location is shown in the upper right corner. **c** This “six-layered” neocortical laminar pattern becomes even more crisp when the distribution of glutamatergic neurons alone is depicted. Inset: a biocytin-filled L2/3 IT (pyramidal) neuron of unknown t-type is shown, which has a typical spiny dendritic tree with an apical dendritic tuft in L1 and an axon running toward the white matter (Preuss, Witte, Staiger; unpublished material). Scale bars, 200 μm for **a** and **b** and 100 μm for the inset. For neuronal cell names, see Tables; for non-neuronal: OGC: oligodendrocyte; OPC: oligodendrocyte precursor cell; PVM: perivascular macrophage; SMC: smooth muscle cell; VLMC: vascular leptomeningeal cell. Modified from BRAIN Initiative Cell Census Network, Nature 2021; with permission

**Fig. 2:**
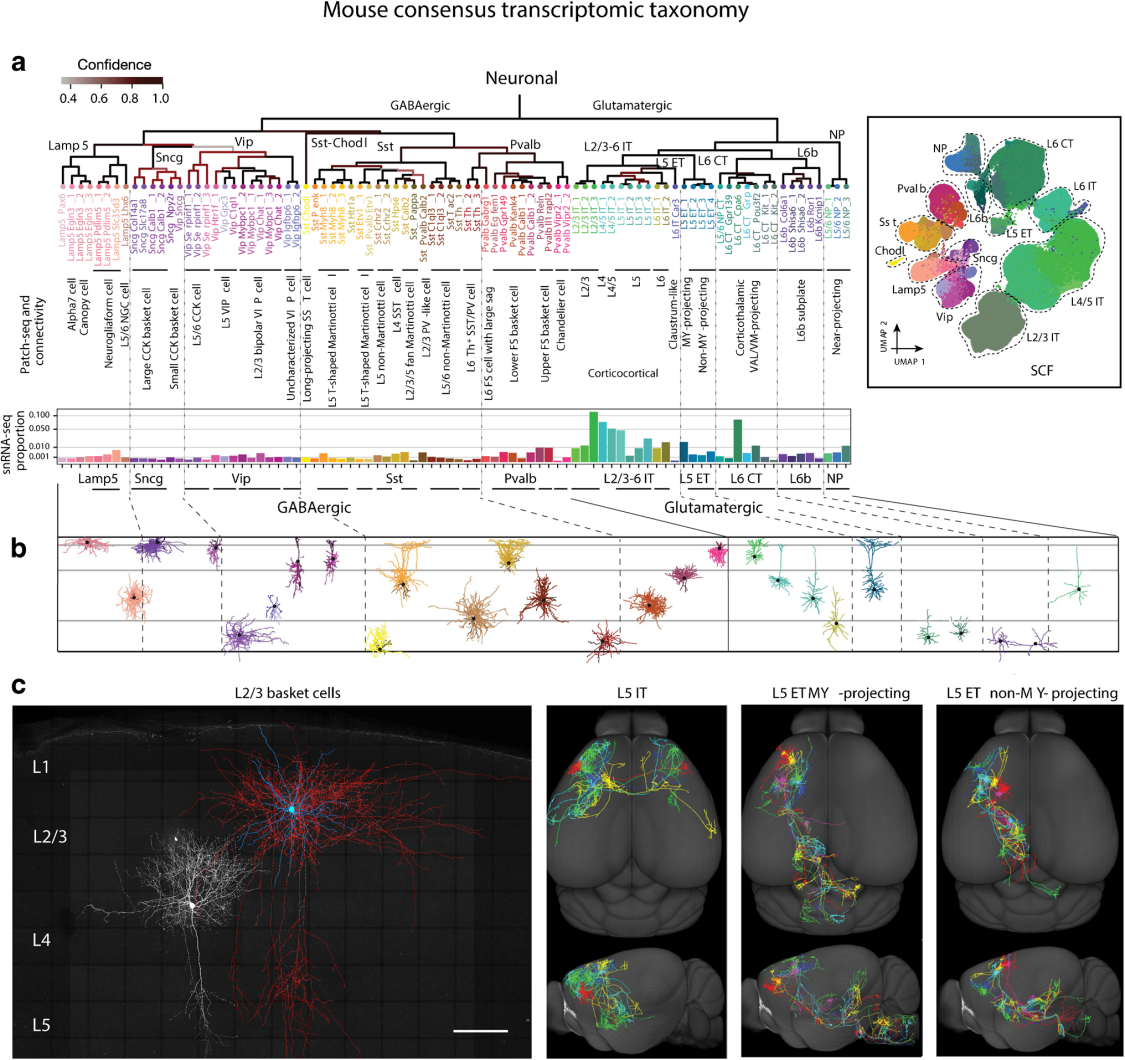
In-depth multimodal account of cortical neuronal cell types. **a** Hierarchical cluster tree showing the consensus transcriptomic taxonomy on mouse neocortical neurons. Major cellular divisions and class and subclass labels are shown above major branches and cluster labels are shown below each leaf node. Using Patch-seq and connectivity studies, many transcriptomic neuron types or subtypes are annotated and correlated with previously established cortical neuron types (see also Table 2). Relative proportions of all cell types are calculated from the snRNA-seq 10x v3 data (bar graphs below cluster tree leaves). The box to the right of the tree presents a UMAP representation of the mouse transcriptomic–epigenomic integrated molecular taxonomy (SCF version). **b** Exemplar candidates for somatodendritic (dark hues, often hidden by axon) and axonal arborizations (brighter hues) of various GABAergic and glutamatergic cell types as derived from Patch-seq studies. **b** Different morphologies of “classical” neocortical cell types. *Left*: Two L2/3 basket cells (fast spiking; not shown) are labeled in primary somatosensory cortex and visualized by high-resolution confocal imaging. For the upper large basket cell, a reconstruction is overlaid in which the somatodendritic compartment is shown in cyan and the axonal compartment in red. Please not the dense local axons with some descending collaterals (Preuss, Witte, Staiger; unpublished material), which also exist in the more compact small basket not superimposed by a reconstruction. Scale bar: 100 μm. *Intermediate*: 5 superimposed L5 IT neurons are visualized on a surface reconstruction of the mouse brain. Their axonal arbors project to ipsilateral and contralateral cortex as well as to striatum. *Right*: Superimpositions of 6 myelencephalon/medulla oblongata-projecting (L5 ET MY-projecting ) versus 6 non-myelencephalon-projecting (L5 ET non-MY-projecting) pyramidal cells are contrasted for their different target regions. Modified from BRAIN Initiative Cell Census Network, Nature 2021; with permission

In the six-layered mouse cortex, excitatory neurons make up the majority of the neuronal population, constituting approximately 80–85% of all neurons. Presently, they are categorized into 9 subclasses based on their soma localization within specific layers (L) and their patterns of projection [2, 17, 42] (Figs. 1 and 2 and Table 1) intratelencephalic projecting (IT), extratelencephalic projecting (ET), near-projecting (NP), and corticothalamic projecting (CT): L2/3 IT, L4/5 IT, L5 IT, L6 IT, Car3 IT, L5 ET, L5/6NP, L6 CT, and L6b. These neuron types can further vary according to their transcription profiles.

**Table 1:** List of some well-described excitatory cell types with main associated features. Please check Table 3 for explanation of molecular markers/gene abbreviations

Cell name	t-type(s)*	Cre-driver line(s)	Electrophysiological feature(s)	Dendritic feature(s)	Hodological feature(s)
L2/3 IT (upper)	*Adamts2; Agmat*	Cux2; Sepw	Regular spiking/adapting/fast adapting	(Modified) pyramidal	Cortico-cortical; cortico-striatal (ipsi- and contralateral)
L4 IT	*Rorb; Rspo1*	Scnn1a; Rorb; Nr5a1	Regular spiking/adapting	Spiny stellate/Star pyramidal/Pyramidal**	Cortico-cortical**
L5 IT	*Whrn; Hsd11b1; Batf3; Col27a1; Deptor*	Tlx3; Tnnt1; Plxnd1	Regular spiking/adapting	(Modified) pyramidal (thin-tufted or short-tufted)	Cortico-cortical; cortico-striatal (ipsi- and contralateral)
L5 ET	*Chrna6; Ptgfr; Krt80; Fam84b*	Rbp4; Sim1; Fezf2 Pvalb	Bursting	Pyramidal (thick-tufted)	Cortico-subcortical
L6 CT	*Ctxn3; Nxph2*	Ntsr1; Tle4	Regular spiking/adapting	(Modified) pyramidal	Cortico-thalamic
L6 IT	*Penk; Col23a1*	Esr2; Osr1	Regular spiking/adapting	Spiny stellate/Star pyramidal/Pyramidal	Cortico-cortical (ipsi- and contralateral)
L6 Car3	*Car3*	Gnb4	Regular spiking/adapting	(Modified) pyramidal	Cortico-cortical (very widespread)
L5/6 NP	*Trhr*	Slc17a8	Regular spiking/adapting	(Modified) pyramidal	Cortico-cortical (restricted range)
L6b	*Mup5; Col8a1*	Ctgf	Regular spiking/adapting	Spiny stellate/star pyramidal	Cortico-cortical

*Mostly taken from Tasic et al. 2018 (primary visual cortex neurons)

**With area-dependent variations

### IT neurons

They were previously called cortico-cortical, cortico-callosal, cortico-striatal, or callosal projection neurons, which are primarily located in layers 2/3, 4, and 5, with fewer numbers found in layer 6. Now, we know that these are not separate neuron types but that the same individual IT neurons project to multiple cortical areas both ipsilaterally and contralaterally through the corpus callosum or the anterior commissure as well as to the striatum [13]. Featuring thin-tufted apical dendrites, L2/3 IT neurons send their major descending interlaminar axonal projection into L5 (Fig. 1C, inset) [11]. They fire sparsely as a consequence of a hyperpolarized resting membrane potential. L4 IT neurons encompass various morphological subclasses, including pyramidal, star-pyramidal, and spiny stellate forms. Interestingly, although primary motor cortex (MOp) lacks a cytoarchitectonic layer 4, neurons resembling those traditionally defined as L4 neurons in sensory cortical regions are also observed [2, 37]. L5 IT neurons are more active than L2/3 IT and have broader projections including more extensive connections to striatum [31]. L6 ITs preferentially establish reciprocal connections with local deep layer neurons [11]. L6 Car3 neurons, having the highest number of targets, possess extensive intracortical axonal projections like other IT neurons, yet lack collateral projections into the striatum [24].

### L5 ET neurons

They are alternatively known as pyramidal tract (PT) or subcerebral projection neurons (SCPN), exhibit thick-tufted dendrites and project to multiple subcortical regions, including the “higher-order” thalamus, midbrain, hindbrain, and the spinal cord (Fig. 2c). They typically display the electrophysiological characteristics of bursts of action potentials leading to a strong impact on their target neurons. Compared to IT neurons, L5 ETs often present with a more depolarized resting membrane potential, lower input resistance, a faster effective membrane time constant, and less spike frequency adaption [1].

### Other neuronal types

L5/6 NP neurons project only sparsely to neighboring regions [32]. Found in layer 6, CT neurons provide feedback projections to the thalamic “relay/first-order” nuclei. In line with their scarcity of local inputs, the majority are notably silent in vivo [11]. L6b subplate neurons reveal projections to L1 within resident and adjacent cortical areas [32], their function in the adult brain remaining enigmatic.

Attempts have been made to classify cortical excitatory neurons using molecular profiles. A varied combination of layer-specific marker genes provided early evidence for the correlation between gene expression and target specificity at the subclass level [13]. Recent advances in single-cell transcriptomics have empowered researchers not only to unveil new markers such as Deptor for L5 IT, Slc17a8 for L5/6 NP [23], Osr1 for L6 IT, and Fam84b for L5 ET [32], but also to discern finer distinctions within the framework of major subclasses (see also Table 1). Then, 19 transcriptomic cell types were identified in primary visual cortex (V1) [31], whereas unsupervised clustering analysis of MERFISH-derived profiles revealed 39 types in primary motor cortex (Mop) [42] (Fig. 1). A combined total of 56 t-types have been discovered in both V1 and the anterior lateral motor cortex (ALM) [32]. The difference is in part due to regional variation but also possibly owns to more extensive cell sampling in ALM. Notably, cortical area differences do not exhibit uniformity across various subclasses, as is shown by L5 ET and L4/5 IT types, which display more pronounced distinctions across areas compared to types in other subclasses. However, it is believed that most types are shared among multiple areas [38]. An apparent difference in t-types between 2 distant areas (primary motor versus primary visual cortex) changes to a more continuous pattern on the global cortical scale. This graded feature is also found across the cortical depth [2, 13, 42]. L2/3-L6 IT types exhibit a continuum marked by gradual changes in gene expression profiles and their positioning within cortical layers. Types with more similar transcriptomes are located at neighboring cortical depths but do not necessarily respect laminar boundaries (Fig. 1c).

Similarly, gradual variation is present in both electrophysiological and morphological properties. In L2/3 of somatosensory and visual cortex, the dendritic tree structure, electrophysiological characteristics and connectional patterns continuously co-vary with pial depth [29, 34]. Results from Patch-seq indicate that properties can exhibit significant variations even within a specific electrophysiological type (e-type) or morphological type (m-type) [9, 26]. Morpho-electric properties exhibit continuous variation across the transcriptomic landscape, suggesting the absence of a clear point to subdivide the cluster. As a result, a one-to-several relationship was observed between t-types like L4-IT–Rspo1 and morpho-electric (me) types, and vice versa, although the integration of both morphological and electrical characteristics exhibited stronger correlations with t-type than examining each modality individually [10].

The exact relationship between projections and t-types remains to be clarified. Nevertheless, a strong alignment exists between major transcriptomic and projection neuron types, as evidenced by the Cre-defined projection mapping strategy [16, 19]. For example, Scnn1a-L4/5 and Ntsr1-L6 neurons exclusively represent L4/5 IT and L6 CT, respectively. Yet, within each subclass, there is no guarantee of a one-to-one correspondence between the finely graded projection pattern and the cell’s t-type, although certain instances like Medulla (MY)-projecting and non-MY-projecting types within L5 ET subclass do map to distinct taxonomic clusters [6]. By contrast, V1 neurons projecting to PM (posteromedial) and V1 neurons projecting to LM (lateromedial) (V1/PM and V1/LM) only form a single genetic cluster [14]. Another noteworthy observation was that neurons of the same t-type may exhibit distinct sets of projection targets specific to their respective home region [24]. This reinforces the notion that transcriptomic classification alone may be insufficient to capture all the heterogeneity within excitatory neuronal types. Therefore, future research endeavors should prioritize the investigation of how molecular, electrophysiological, morphological characteristics, and projection targets interact when defining cell types.

## Current status of inhibitory cell type classification

While GABAergic interneurons comprise only 15–20% of cortical neurons, they exhibit the most extensive diversity in terms of morphology, electrophysiology, and neurochemical characteristics. By the 2005 Petilla Convention, a consensus nomenclature was put forth to improve the characterization of interneurons, yet failed to live up to expectations. Frequently, the same name was assigned to neurons with differing morphologies, and various terminologies were inconsistently adopted across different laboratories to describe the same cell classification [5, 25]. Decades later, single-cell transcriptomics has undertaken the challenge of resolving this long-standing issue and now stands as the anchor for defining cell types. Although similar to excitatory neurons, a degree of continuous variation exists within each t-type and the correspondence between t-types and electrophysiological or morphological features is not strictly one-to-one [9, 26, 32], 28 morphological/electrophysiological/transcriptomic types (met-types) of cortical interneurons with congruent properties were identified in V1 [9]. Here, we will highlight several widely accepted types, ignoring putative projection neurons and the still poorly studied Sncg neurons (Figs. 1, and 2 and Table 2).

**Table 2:** List of some well-described inhibitory cell types with main associated features. Please check Table 3 for explanation of molecular markers/gene abbreviations

Cell name	Classical molecular marker(s)*	t-type(s)	Electrophysiological feature(s)	Dendritic feature(s)	Axonal feature(s)
Axo-axonic (Chandelier) cell	Parvalbumin	*Vipr2*	(Often) fast spiking	Multipolar	Axonal cartridges targeting the axon initial segment of excitatory cells
Basket cell	Parvalbumin	*Calb1; Reln*	Fast spiking	Multipolar	Axonal arbors extending spherically around the somatodendritic domain
Martinotti cell	Somatostatin	*Calb2; Etv1*	Adapting; bursting	Multipolar to bitufted (not so sparsely spiny)	Ascending axonal arbor targeting layer 1 (and other layers to a varying degree)
Non-Martinotti cell	Somatostatin	*Crhr2; C1ql3*	Non-adapting; quasi-fast spiking	Multipolar to bitufted	Axonal arbor not targeting layer 1 but showing layer 4 preference
Inhibitory interneuron-specific interneuron	Vasoactive intestinal polypeptide	*Mybpc1; Chat*	Adapting; irregular spiking; bursting	Bipolar-bitufted	Descending axonal arbor (often columnar)
Neurogliaform cell	Neuron-derived neurotrophic factor; neuropeptide Y	*Lamp5; Pdlim5*	Adapting; late-spiking (if neuropeptide-Y co-expressing)	Multipolar	Dense spherical axon with volume transmission

*Also used as Cre-(or other recombinase-specific) driver lines

**Table 3:** List of all the molecular markers mentioned in the text

Gene symbol	Description	Function
PV	Parvalbumin	Calcium-binding protein, precise function still unknown. Can be up- or downregulated according to previous neuronal activity history
SST	Somatostatin	Inhibits the release of numerous secondary hormones by binding to high-affinity G-protein-coupled somatostatin receptors, affects rates of neurotransmission
VIP	Vasoactive intestinal peptide	Belongs to the glucagon family, causes vasodilation, increases glycogenolysis, and lowers arterial blood pressure
Lamp5	Lysosomal-associated membrane protein family member 5	Involved in establishment of protein localization to lysosomal organelles, plays a role in short-term synaptic plasticity
Sncg	Synuclein gamma	Belongs to the synuclein family, plays a role in neurofilament network integrity. May be involved in modulating axonal architecture during development and in the adult
Adamts2	ADAM metallopeptidase with thrombospondin type 1 motif 2	Belongs to the ADAMTS protein family, cleaves the propeptides of type I and II collagen prior to fibril assembly
Agmat	Agmatinase	Hydrolyzes linear guanidino acids to form urea and the corresponding amines
Cux2	Cut-like homeobox 2	Transcription factor involved in the control of neuronal proliferation and differentiation in the brain. Regulates dendrite development and branching, dendritic spine formation, and synaptogenesis in cortical layers II-III
Sepw	Selenoprotein W	Glutathione (GSH)-dependent antioxidant involved in the protection of neurons from oxidative stress during neuronal development
Rorb	RAR-related orphan receptor B	Member of the NR1 subfamily of nuclear hormone receptors, NA-binding protein that can bind to hormone response elements, controls patterning of neocortical neurons during development, acts in a dose-dependent manner to regulate barrel formation upon innervation of layer IV neurons by thalamocortical axons
Rspo1	R-Spondin 1	Activator of the canonical Wnt signaling pathway by acting as a ligand for LGR4-6 receptors
Scnn1a	Sodium channel epithelial 1 subunit alpha	Component of non-voltage-gated, amiloride-sensitive sodium channel, controls fluid and electrolyte transport across epithelia in many organs
Nr5a1	Nuclear receptor subfamily 5 group a member 1	Transcriptional activator, essential for sexual differentiation
Whrn	Whirlin	Functions in the organization and stabilization of stereocilia elongation and actin cytoskeletal assembly, involved in hearing and vision as member of the USH2 complex
Hsd11b1	Hydroxysteroid 11-beta dehydrogenase 1	Enzyme controlling the reversible conversion of biologically active glucocorticoids (such as cortisone to cortisol) in the presence of NADP(H)
Batf3	Basic leucine zipper ATF-like transcription factor 3	Member of the basic leucine zipper protein family, functions as a transcriptional repressor when heterodimerizing with JUN
Col27a1	Collagen type XXVII alpha 1 chain	Member of the fibrillar collagen family, plays a role during the calcification of cartilage and the transition of cartilage to bone
Deptor	DEP domain containing mTOR interacting protein	Negative regulator of the mTORC1 and mTORC2 complexes, leading to a negative regulation of cell size
Tlx3	T cell leukemia homeobox 3	DNA-binding nuclear transcription factor
Tnnt1	Troponin T1, slow skeletal type	Tropomyosin-binding subunit of troponin, early molecular biomarker of stress response
Plxnd1	Plexin D1	Cell surface receptor for SEMA4A and for class 3 semaphorins, enables protein domain specific binding activity, plays an important role in ensuring the specificity of synapse formation
Chrna6	Cholinergic receptor nicotinic alpha 6 subunit	Alpha subunit of neuronal nicotinic acetylcholine receptors, involved in neurotransmission
Ptgfr	Prostaglandin F receptor	Receptor for prostaglandin F2-alpha, activity mediated by G proteins which activate a phosphatidylinositol-calcium-based second messenger system
Krt80	Keratin 80	Intermediate filament protein responsible for the structural integrity of epithelial cells, involved in cell differentiation
Fam84b	Family with sequence similarity 84 member B	Involved in cell morphogenesis and cell motility
Rbp4	Retinol binding protein 4	Retinol-binding protein that mediates retinol transport in blood plasma
Sim1	SIM BHLH transcription factor 1	Transcriptional factor that is involved in certain dysmorphic features and abnormalities of brain development
Fezf2	FEZ family zinc finger 2	Transcription repressor required for the specification of corticospinal motor neurons and other subcerebral projection neurons, plays a role in layer and neuronal subtype-specific patterning of subcortical projections and axonal fasciculation
Ctxn3	Cortexin 3	Integral component of membrane, associated with schizophrenia
Nxph2	Neurexophilin 2	Signaling molecule that resembles neuropeptides and acts by binding to alpha-neurexins
Ntsr1	Neurotensin Receptor 1	G-protein coupled receptor for the tridecapeptide neurotensin
Tle4	TLE family member 4, transcriptional corepressor	Transcriptional corepressor that binds to a number of transcription factors, inhibits the transcriptional activation mediated by PAX5, CTNNB1 and TCF family members in Wnt signaling
Penk	Proenkephalin	Preproprotein that is proteolytically processed to generate multiple protein products, including the pentapeptide opioids Met-enkephalin and Leu-enkephalin, which are stored in synaptic vesicles, then released into the synapse where they bind to mu- and delta-opioid receptors
Col23a1	Collagen type XXIII alpha 1 chain	Member of the transmembrane collagens
Esr2	Estrogen receptor 2	Nuclear hormone receptor, binds estrogens and activates expression of reporter genes containing estrogen response elements in an estrogen-dependent manner
Osr1	Odd-skipped-related transcription factor 1	Transcription factor that enables sequence-specific double-stranded DNA binding activity, involved in negative regulation of transmembrane ion transporter activity
Car3	Carbonic anhydrase 3	Reversible hydration of carbon dioxide
Gnb4	G protein subunit beta 4	Modulator or transducer in various transmembrane signaling systems, beta and gamma chains are required for the GTPase activity, for replacement of GDP by GTP, and for G protein-effector interaction
Trhr	Thyrotropin -releasing hormone receptor	Receptor for thyrotropin-releasing hormone, triggers activation of the phosphatidylinositol (IP3)-calcium-protein kinase C (PKC) pathway upon ligand binding
Slc17a8	Solute carrier family 17 member 8	Multifunctional transporter that transports L-glutamate as well as multiple ions such as chloride, sodium and phosphate
Mup5	Major urinary protein 5	Enables odorant binding activity
Ctgf	Cellular communication network factor 2	Major connective tissue mitoattractant secreted by vascular endothelial cells, mediates heparin- and divalent cation-dependent cell adhesion
Col8a1	Collagen type VIII alpha 1 chain	Macromolecular component of the subendothelium
Ndnf	Neuron-derived neurotrophic factor	Secretory protein, promotes neuron migration, growth, and survival as well as neurite outgrowth
Npy	Neuropeptide Y	Neuropeptide that functions through G protein-coupled receptors to inhibit adenylyl cyclase, activates mitogen-activated protein kinase, regulates intracellular calcium levels, and activates potassium channels
Vipr2	Vasoactive intestinal peptide receptor 2	Receptor for vasoactive intestinal peptide, whose activity is mediated by G proteins which activate adenylyl cyclase
Calb1	Calbindin 1	Calcium-binding protein, buffers entry of calcium upon stimulation of glutamate receptors
Reln	Reelin	Extracellular matrix serine protease that plays a role in layering of neurons in the cerebral cortex and cerebellum, regulates microtubule function in neurons and neuronal migration
Calb2	Calbindin 2	Intracellular calcium-binding protein (also known as calretinin), plays a role in diverse cellular functions, including message targeting, intracellular calcium buffering and thus modulates neuronal excitability
Etv1	ETS variant transcription factor 1	Member of the ETS family of transcription factors, targets genes that modulate biological processes like cell growth, angiogenesis, migration, proliferation and differentiation
Crhr2	Corticotropin-releasing hormone receptor 2	G-protein coupled receptor for CRH (corticotropin-releasing factor), UCN (urocortin), UCN2 and UCN3
C1ql3	Complement C1q Like 3	Member of the complement protein family, enables identical protein binding activity, acts upstream of or within regulation of synapse organization
Mybpc1	Myosin binding protein C1	Member of the myosin-binding protein C family, modulates muscle contraction
Chat	Choline O-acetyltransferase	Enzyme which catalyzes the biosynthesis of the neurotransmitter acetylcholine
Pdlim5	PDZ and LIM domain 5	Contributes to the regulation of dendritic spine morphogenesis in neurons
Myh8	Myosin heavy chain 8	Member of the class II or conventional myosin heavy chains, functions in skeletal muscle contraction
Hpse	Heparanase	Endoglycosidase that cleaves heparan sulfate proteoglycans into heparan sulfate side chains and core proteoglycan to permit cell movement through remodeling of the extracellular matrix
Itga4	Integrin subunit alpha 4	Associates with a beta 1 or beta 7 subunit to form an integrin that plays a role in cell motility and migration
Ptprt	Protein tyrosine phosphatase receptor type T	Involved in both signal transduction and cellular adhesion
Pkp2	Plakophilin 2	Regulates focal adhesion turnover, resulting in changes in focal adhesion size, cell adhesion, and cell spreading
Lmo1	LIM domain only 1	Transcription factor involved in gene regulation within neuronal lineage cells, potentially by direct DNA binding or by binding to other transcription factors
Fam159b	Family with sequence similarity 159 member B	Integral component of membrane
Cck	Cholecystokinin	Neuropeptide and gut hormone that regulates pancreatic enzyme secretion and gastrointestinal motility. Sulfated form of cholecystokinin-8 modulates neuronal activity in the brain
Lhx6	LIM homeobox 6	Transcription factor required for the expression of a subset of genes involved in interneuron migration and development
Chrna7	Cholinergic receptor nicotinic alpha 7 subunit	Subunit of neuronal nicotinic receptors that mediate fast signal transmission at synapses
Fam19a1	Family with sequence similarity 19 Member A1	Member of the TAFA family, functions as brain-specific chemokine or neurokine
Tmem182	Transmembrane protein 182	Integral component of membrane, negatively regulating myogenesis and skeletal muscle regeneration via its association with ITGB1
Nmbr	Neuromedin B receptor	G protein-coupled receptor that binds neuromedin B, plays a role in neuronal responses and the regulation of cell growth

The initial branches of the hierarchical t-type tree diagram distinguish between excitatory glutamatergic and inhibitory GABAergic classes, probably underlining their distinct developmental origins in the embryonic pallium and subpallial regions (Fig. 1a). The GABAergic branch further separates into 5 subclasses originating from the medial and caudal ganglionic eminence (MGE and CGE) based on marker molecules: parvalbumin (PV) and somatostatin (SST) in MGE and vasoactive intestinal polypeptide (VIP), Lamp5, and Sncg in CGE [32, 42].

Within the PV population, two well established anatomical subtypes stand out: fast-spiking basket cells (PV-BCs) and chandelier cells (ChCs). BCs target the perisomatic domain of pyramidal cells [7]. Contributing significantly to the cortical excitation-inhibition balance, they are credited with various perception-related functions, such as the generation of gamma-band oscillations of neuronal ensembles, as well as modulating gain control [30]. Found in cortical layers 2–6, layer 4 PV-BCs are mainly mapped to Pvalb Reln [9], exhibiting local axonal arborizations. However, PV-BCs in supra- and infragranular layers possess more extensive axonal projections which extend into neighboring columns and layers (Fig. 2c) [33]. ChCs (also called axo-axonic cells) are characterized by axonal arbors that resemble the candlesticks of a chandelier and specialize in targeting the axon initial segment of pyramidal cells, imposing an even stronger output control on them than PV-BCs. ChCs belong to the Pvalb Vipr2 t-type [9, 26].

The SST subclass encompasses diverse cell types, including Martinotti cells (MCs) and non Martinotti cells (NMCs). MCs are considered to primarily target distal apical dendrites of pyramidal cells through an extensive axonal arbor in layer 1, playing a vital role in feedback inhibition [21]. However, they also possess extensive axonal arborization outside of L1, the targeting of which is still unknown. Besides SST-Calb2 MCs in layer 2/3, 2 types of MCs exist in layer 5. While L5 T-shaped MCs primarily innervate layer 1 apical tufts, exhibiting low-threshold spiking, L5 fanning-out MCs innervate layer 2/3 and the lower half of layer 1 with adapting firing patterns [22]. They are defined by Myh8 and Etv1 expressions [9, 11, 35], respectively. Quasi-fast-spiking L4/L5A SST-Hpse [20, 26, 35] NMCs, with axon extensively targeting L4, relay information from the lemniscal thalamus [22]. The L5/6 NMCs possess, next to local axon collaterals, an ascending projection mostly targeting L4. They are associated with SST-Crhr2 and SST-C1ql3 t-types [9, 26].

The majority of VIP interneurons preferentially target other GABAergic subclasses, notably SST cells, consequently disinhibiting local pyramidal neurons [15]. They receive inputs from higher-order cortical areas and neuromodulatory inputs from subcortical regions [21]. Bipolar/bitufted VIP interneurons feature vertically oriented dendrites that extend into layer 1. Layer 2/3 VIP cells possess axonal arborizations that extend downward to the layer 6/white matter boundary [22], with Vip-Rspo1-Itga4 displaying regular spiking and Vip-Ptprt-Pkp2 (mostly associated with Chat^+^ cells) displaying irregular spiking [9, 10]. In contrast, Vip-Lmo1-Fam159b cells residing in layers 5 and 6 exhibit a more limited pattern of axonal arborizations, mainly confined to these layers [9]. Small basket cells, another (assumed) subtype of VIP interneuron, exhibit a predominantly localized axonal arbor and are characterized by the expression of CCK. These neurons possess small soma and are primarily distributed in layer 2/3. Unlike other VIP interneuron subtypes, they establish perisomatic basket terminals akin to PV-BCs [33]. There is some debate whether the Sncg subclass (at least partially) corresponds to VIP/CCK cells [32] or whether they form separate subclasses. They display regular, adapting firing patterns.

Neurogliaform cells (NGCs) with multipolar dendritic arbors and the notable feature of a highly dense perisomatic axonal arborization correspond to several Lamp5 types. Found in every layer, being especially prominent in layer 1, they are (partially) known for their late-spiking behavior, utilizing volume transmission with many thousands of boutons in their axonal arbor. Their activation of postsynaptic metabotropic GABA_B_ receptors potentially serves as a source of slow inhibition [30]. Additionally, they exhibit gap junctional coupling with other inhibitory neuron types [31]. Intriguingly, the Lamp5 Lhx6 type (associated with deep L5/L6 NGCs) is believed to originate from the MGE, distinguishing it from all other Lamp5 types [32]. Layer 1 harbors two other subtypes: Alpha7 cells and canopy cells. Both of these subtypes display a non-late-spiking adapting firing pattern. Alpha7 cells are aligned with Lamp5-Chrna7 markers and are characterized by a prominent hyperpolarizing sag, along with axon collaterals that extend into L5A. Conversely, canopy cells, identified as Lamp5-Fam19a1-Tmem182 types, feature horizontally elongated axonal arbors primarily confined to the upper half of L1 [3, 27].

The question of whether neuronal classification can be based on underlying biological mechanisms or remains phenomenological has been a longstanding debate. However, recent evidence has demonstrated that distinct subtypes perform specific circuit functions, implying that these subtypes indeed exist rather than being a numbers game. For example, SST-Calb2 MCs selectively innervate L5-PT, while SST-Nmbr NMCs predominantly target L5-IT neurons [35]. During whisking, the activity of L2/3 MCs decreased. Conversely, L4-6 NMCs became more active [18]. Another interesting case was the subtypes in V1, of which the modulation state was observed to be associated with their positions along the main axis of transcriptomic variation (transcriptomic principal component 1). Subtypes at the negative end of the axis were most prominently active during synchronized states, while those that occupied the most positive end were most active during desynchronized and running states [3]. The notable variations of modulation state among subtypes seem to be another indication of continuous transcriptomic variation rather than discrete subtypes. The smoothly varying neuronal activity along the transcriptomic continuum offers an alternative viewpoint on the knotty situation of continuity within/across t-types. We believe there is an urgent need for more research to establish connections between cell type classification and the functional characteristics of their cortical area-specific circuits.

## Conclusions and outlook

The journey from intuitive morphological criteria to cutting-edge transcriptomic profiling to multimodal classification represents a remarkable evolution in our quest to unravel the intricacies of the brain’s cellular landscape [12]. This transformation has not only expanded our understanding of neuronal diversity but also paved the way for more precise and comprehensive cell classifications.

One of the key strategies employed in this pursuit is the integration of multiple criteria for classification, a shift from flat to hierarchical systems, which inherently incorporates relationships between types into the classification and offers a versatile approach for adapting the system in response to new information [41]. Nevertheless, challenges persist in addressing the overlay of discrete cell type distinctions with graded properties. Though neurons feature discrete, non-overlapping branches at the family level, they have the capacity to create continuous transcriptomic and morpho-electrical landscapes within these families [2, 9, 26, 38, 42]. Finding a ‘sweet spot’ between splitter and lumper perspectives requires theoretical breakthrough for a principled framework on conceptualization of cell variability, which should to the largest extent possible reflect the functional consequences associated with different cell rather than reducing the classification process to a subjective exercise. Flexibility is paramount; the framework must adapt to the dynamic nature of individual cell entities, recognizing that these entities are not static but evolving components of intricate biological systems. In essence, the emphasis should not solely be on the sheer quantity of identified cell types but on the meaningful understanding of how these cell types carry relevance in terms of their specific contributions to information processing and network dynamics. While acknowledging the significance of the transcriptomic signature, it should primarily function as the bedrock for unveiling specific functional properties and establishing a causal link between gene expression and cellular function. Caution is urged against overprioritizing transcriptomics for classification purposes. Instead, this perspective encourages a more purpose-driven methodology in single-cell transcriptomics. It advocates for a thoughtful and context-driven approach, which ensures that the classification process is not a detached enumeration but remains intimately connected to the understanding of how molecular characteristics manifest in observable and functionally relevant features of neurons.

Furthermore, the road ahead involves the development of a unified nomenclature for neural cell types and fostering collaboration among research groups and analysis platforms. Initiatives like the Brain Initiative Cell Census Consortium (BICCC) [2, 17] embody these goals, aiming to generate a census of cell types and facilitate cross-areal and cross-species comparisons. These collective efforts are designed to provide open-access resources and tools that can benefit the broader research community.

The classification will aid in precise cell targeting and unlock invaluable insights into the developmental mechanisms driving neuronal identity and diversity also in the cerebral cortex, a task that was already successfully achieved in areas like the hypothalamus [8] or brainstem/spinal cord [4]. With the help of different recombinase-specific driver lines that replicate the activity of crucial transcription factors involved in specification and differentiation [11], fate-mapping of biologically important subtypes of neurons will enable the exploration of spatiotemporal determinants shaping their developmental trajectories. This could potentially be the linchpin for programming and reprogramming cortical neurons, offering innovative therapeutic approaches for diseases that predominantly affect specific neuronal populations.

Future efforts must explore additional dimensions, including local connectivity, multi-omics, in vivo functional characterization, brain states, network dynamics on shorter and longer time scales, and role of neuromodulators. Breakthrough techniques like automated morphological reconstruction and bona-fide measurements of dynamic changes, instead of snapshots of a cell, will also open new frontiers in research. This journey into the multidimensional realm holds the potential to usher in a new era of data-driven cell classification and a deeper understanding of the brain.

## Data Availability

Not applicable
